# Obstructive spirometry patterns in young adults with asthma and rhinitis from rosario, Argentina

**DOI:** 10.1186/1939-4551-8-S1-A119

**Published:** 2015-04-08

**Authors:** Ledit Ardusso, Luciano Rovetto, Mariela Formaggio, Matias Duarte, Guillermo Mujica, Nora Figueroa, Paula Daneri, Rut Aguero, Jorge Molinas

**Affiliations:** 1 National University of Rosario, Argentina

## Background

Asthma and allergic rhinitis have become a growing public health problem. The airflow obstruction, pathophysiological event that frames the bronchial asthma is usually determined by spirometry. Changes in expiratory flow in asthma are well known but there is controversy with these results in allergic rhinitis. The objective of this project is to compare spirometry values of young adults without respiratory disease with individuals of the same age group who have: allergic rhinitis without bronchial asthma or bronchial asthma without allergic rhinitis.

## Methods

Were studied (randomly selected) 279 students (185 women and 94 men) aged between 18 and 30 years (x = 21.68 ± 2.29) who were in the first three years of the Medical Sciences Faculty National University of Rosario, Argentina, during 2014. Students gave their informed consent to undergo effort spirometry with ATS technique (Multispiro III spirometer), recording the values of forced vital capacity (FVC), forced expiratory volume in one second (FEV1) and forced expiratory flow at 25-75% of the pulmonary volume (FEF25-75). The statistical analysis of the information was conducted using EPI INFO.

## Results

The current frequency of rhinitis and asthma in the sample was 54.1% and 17.2% respectively. 40.5% of individuals did not show any pathology, 42.3% suffered only current rhinitis and 5.4% only bronchial asthma. Was found significant decrease in FEF25-75 (p=0.01) in subjects suffering from rhinitis alone (3.18±1.35 liters) than individuals without respiratory disease (4.88±1.51 liters). Furthermore, the known decrease in spirometric parameters between subjects with asthma and those who did not have any respiratory disease was observed (TIFF: 82.48±12.86% vs 92.03±7.43%, p=0.005; FEV1: 2.95±0.68 vs 3.76±0.96 liters, p=0.02; FEF25-75: 3.18±1.35 vs 4.88±1.51 liters, p=0.00006). No significant differences were found in FVC.

## Conclusions

This work find the known association between decline in FEV1, index TIFF and FEF25-75 in subjects with bronchial asthma when compared with healthy individuals, but also showed a significantly decreased FEF25-75 only in subjects suffering from allergic rhinitis when compared with healthy individuals. These data are consistent with some studies suggesting that small airway disease, related with a reduction in FEF25-75 may be a marker for early allergic or inflammatory involvement of the small airways in subjects with allergic diseases and no asthma. These data show the need to continue these studies with larger numbers of individuals and adjusting variables involved.

